# Can Male Circumcision Have an Impact on the HIV Epidemic in Men Who Have Sex with Men?

**DOI:** 10.1371/journal.pone.0102960

**Published:** 2014-07-30

**Authors:** Steven M. Goodreau, Nicole B. Carnegie, Eric Vittinghoff, Javier R. Lama, Jonathan D. Fuchs, Jorge Sanchez, Susan P. Buchbinder

**Affiliations:** 1 University of Washington, Seattle, Washington, United States of America; 2 Harvard School of Public Health, Boston, Massachusetts, United States of America; 3 University of California San Francisco, San Francisco, California, United States of America; 4 Asociación Civil Impacta Salud y Educación, Lima, Perú; 5 San Francisco Department of Public Health, California, United States of America; Rollins School of Public Health, Emory University, United States of America

## Abstract

**Background:**

Three trials have demonstrated the prophylactic effect of male circumcision (MC) for HIV acquisition among heterosexuals, and MC interventions are underway throughout sub-Saharan Africa. Similar efforts for men who have sex with men (MSM) are stymied by the potential for circumcised MSM to acquire HIV easily through receptive sex and transmit easily through insertive sex. Existing work suggests that MC for MSM should reach its maximum potential in settings where sexual role segregation is historically high and relatively stable across the lifecourse; HIV incidence among MSM is high; reported willingness for prophylactic circumcision is high; and pre-existing circumcision rates are low. We aim to identify the likely public health impact that MC interventions among MSM would have in one setting that fulfills these conditions—Peru—as a theoretical upper bound for their effectiveness among MSM generally.

**Methods and Findings:**

We use a dynamic, stochastic sexual network model based in exponential-family random graph modeling and parameterized from multiple behavioral surveys of Peruvian MSM. We consider three enrollment criteria (insertive during 100%, >80% or >60% of UAI) and two levels of uptake (25% and 50% of eligible men); we explore sexual role proportions from two studies and different frequencies of switching among role categories. Each scenario is simulated 10 times. We estimate that efficiency could reach one case averted per 6 circumcisions. However, the population-level impact of an optimistic MSM-MC intervention in this setting would likely be at most ∼5–10% incidence and prevalence reductions over 25 years.

**Conclusions:**

Roll-out of MC for MSM in Peru would not result in a substantial reduction in new HIV infections, despite characteristics in this population that could maximize such effects. Additional studies are needed to confirm these results for other MSM populations, and providers may consider the individual health benefits of offering MC to their MSM patients.

## Introduction

Three groundbreaking trials have demonstrated the prophylactic effect of male circumcision (MC) for HIV acquisition among heterosexuals [1]–[3]. Since that time, extensive efforts to roll out elective MC among populations in sub-Saharan Africa, where heterosexual transmission predominates, have occurred [4]. MC for men who have sex with men (MSM-MC) has not seen the same focus, for multiple reasons. For one, no trial has occurred demonstrating the per-act or per-time-unit effectiveness of MSM-MC during insertive anal sex. More fundamentally, the ability for MSM to be sexual-role-versatile may nullify much of MC's protective effect. That is, an MSM who elects circumcision will have the same acquisition risk from each act of unprotected receptive anal intercourse (URAI) as an uncircumcised MSM, and the same probability of transmitting as an uncircumcised man does during unprotected insertive anal intercourse (UIAI; this and other acronyms listed in Table 1). Even if MC has the same proportional per-act protective effect for UIAI as for unprotected insertive vaginal intercourse (UIVI), the population-level impact of an MSM-MC intervention would depend on the proportion of MSM taking the insertive role exclusively during UAI, as well as the proportions who are versatile or exclusively receptive. There is evidence that these proportions vary among MSM populations globally for historical and cultural reasons [5]–[8]. One region where sexual role segregation may be relatively common is Latin America, where it is a long-standing aspect of MSM sexual identity [9]–[12]. In Peru, for example, sexual role is tightly interwoven with identity, and correlates with income, education and behavioral bisexuality [13]. MSM-MC would be expected, then, to have the largest public health impact on lowering new infections in a setting like Peru, given relatively high sexual role segregation, along with high HIV incidence among MSM (estimated at 3.5% per year [14]) and a very low prevalence of neonatal male circumcision (5% in one estimate [15]).

**Table 1:** Abbreviations used frequently in the text.

MSM	Men who have sex with men
MC	Male circumcision
MSM-MC	Male circumcision for MSM
AI	Anal intercourse
UAI	Unprotected (i.e. condom-less) anal intercourse
UIAI	Unprotected insertive anal intercourse
URAI	Unprotected receptive anal intercourse
UIVI	Unprotected insertive vaginal intercourse
RCS	Role category switching: the practice of moving among being exclusively insertive, exclusive receptive or versatile over the life course
HRS	High role segregation: a modeled scenario based on data from the HPTN-039 study

As summarized in two meta-analyses and one review [16]–[18], numerous observational studies have found a non-significant (but usually protective) effect of MSM-MC generally; when limited to predominantly insertive MSM, most have found a protective effect, some with statistical significance. A 2008 meta-analysis estimated an odds ratio (OR) for HIV infection by circumcision status of 0.86 for MSM overall (95% CI 0.65–1.13) and 0.71 for predominantly insertive MSM (95% CI 0.23–2.22) [16]. A subsequent 2011 meta-analysis found an identical OR for MSM overall (0.86, 95% CI 0.70–1.06) and a much lower OR for predominantly insertive MSM (0.27% CI 0.17–0.44) [18]. The last measure was largely driven by a single South African study (76.9% of the meta-analysis weight); studies from resource-high settings averaged higher ORs [19]. One analysis with a predominantly (75%) Peruvian MSM sample obtained point estimates (using relative risks, RR) similar to both meta-analyses for all MSM (0.84, 95% CI 0.50–1.42) and to the latter meta-analysis for predominantly insertive MSM (0.31, 95% CI 0.06–1.51), in this case defined as men insertive ≥60% of the time with recent male partners [20]. The overall pattern, then, suggests that resource-limited settings and/or settings with strong historical patterns of sexual role segregation may show stronger effects in observational studies. Unfortunately, definitions for “predominantly insertive” varied across study.

However, the OR or RR of HIV infection by circumcision status alone does not tell us the potential public health impact of an MSM-MC intervention, given the potential for “spillover”—the fact that a given circumcision might prevent not only transmission to an insertive man, but also subsequent onward transmissions to his receptive partners. Estimating this potential population-level impact thus requires mathematical modeling. Two such studies have considered this question, both for high-resource settings [21], [22]. Both found the overall long-term population-level impact of a hypothetical MSM-MC intervention to be modest (∼5% decrease in prevalence for explored scenarios), although the one paper conducting an explicit cost-benefit analysis found MSM-MC to be likely cost-effective and possibly cost-saving [21]. No published model has considered such an intervention in resource-limited settings.

Multiple studies have considered MSM's willingness to consider prophylactic MC in a host of settings. Willingness is generally low (∼10%) in high-resource settings [23], [24], but higher (∼30%) in some resource-limited Asian settings [25]–[27]. One San Francisco study found moderate willingness (20%) among the target population (HIV-negative, predominantly insertive, uncircumcised MSM who have UAI); however, the proportion of the MSM sample found in the target population was so small (3.7% or n = 15) that the study anticipated limited public health benefits for MSM-MC [28]. In contrast, unpublished results from Peru and Ecuador suggest that around half (54.3%) of uncircumcised MSM would be willing to participate in an MSM-MC trial [29]. This, combined with higher rates of sexual role segregation and lower rates of circumcision in these settings, suggest that it may be possible for an MSM-MC intervention in this community to generate significant public health impact through both direct and indirect (spillover) effects. Other areas that share some or all of these characteristics include the remainder of the Andean region, as well as much of Central America [30]. The literature discussed above suggests that South Africa (and perhaps elsewhere in Southern Africa) and some areas of South East or East Asia may match these key criteria as well.

In this paper, we adopt a stochastic network-based model of HIV transmission among MSM developed for the Prevention Umbrella for MSM in the Americas (PUMA) Project, parameterized using behavioral data from multiple studies, to consider the impacts of MSM-MC interventions in Peru. We consider this as a reasonable approximation to a best-case scenario for MSM-MC worldwide, given the set of conducive conditions there. Intervention scenarios are defined by two characteristics—inclusion criterion (insertive during recent acts of UAI either 100%, >80% or >60% of the time), and uptake (25% or 50% of eligible MSM). We conduct sensitivity analyses on these models using both different data sources for the prevalence of sexual role categories (exclusive insertivity, versatility, exclusive receptivity) and on the frequency of switching among sexual role categories over time. Finally, to help contextualize the ORs obtained in observational studies, and to justify some of our modeling assumptions, we estimate the ORs for HIV status by circumcision status that would be obtained if one sampled either all MSM or predominantly insertive MSM from our modeled population.

## Methods

The PUMA modeling framework has been described in detail elsewhere [7]; we adapt “Model 2” from this previous work. In brief, it is a stochastic network-based model with an initial population size of 10,000 MSM; in the Peru version, men are distinguished by age, circumcision status, infection status, sexual role preference, time since infection, diagnosis status, viral load, treatment status (including adherence), and propensity for casual UAI. Sexual role preference includes exclusive insertives; exclusive receptives, and versatiles; versatile men are further distinguished by an insertive propensity, drawn from a (0,1) uniform distribution. We track both main partnerships and casual contacts, each using exponential random graph modeling [31]–[34], with relational and UAI probabilities determined by age, diagnosis status, casual UAI propensity, AIDS status, sexual role, and existence of other ongoing relationships. Each day, relationships may form and/or break; UAI and potentially transmission occur; viral load is updated; and men may enter the population, get tested, initiate treatment, stop treatment, and/or die of AIDS or natural causes. Under the intervention model, men also may opt for circumcision. Behavioral parameters are derived from multiple studies, predominantly the Peru 2008 Sentinel Surveillance [35] and the baseline of HPTN-039 (a trial of daily oral HSV-2 suppressive therapy with acyclovir to prevent HIV acquisition) [36], and are summarized in the Appendix in [15].

Table 2 details the scenarios explored, including the proportion of HIV-negative MSM receiving adult MC under each intervention scenario. All of our interventions target predominantly insertive MSM, for which we model three definitions: men with propensities towards insertivity during UAI of 100%, >80% or >60%. Men are eligible if they meet this definition, are uncircumcised, and are HIV-negative. For each definition of predominantly insertive, we consider two uptake levels (25%, 50%) among eligible men. Adult men who are eligible and willing to uptake at rollout do so then, regardless of age; subsequently, men opt for circumcision as they enter the sexually active population or first meet the definition of predominantly insertive MSM, with probability equal to uptake. We assume that healing lasts 30 days [37]. With no numerical estimates at the time for increased per-act risk during healing, nor for realized UAI frequency reduction while healing in these populations, we arbitrarily assumed values of 30% and 50% respectively, and note that basic sensitivity analyses showed results to be highly insensitive to these choices given the short duration of healing. Throughout, we assume the same proportional reduction in transmission potential (∼60%) from MC for UIAI as for UIVI [1]–[3].

**Table 2:** Scenarios explored.

	Inclusion criteria for intervention	Uptake of intervention among eligible men	Expected distribution of sexual role at a cross-section in time			Mean frequency of role switching	Comparison model for assessing intervention impact	% of HIV-negative MSM receiving adult MC
			Excl. insertive	Versatile	Excl. receptive			
Main baseline	–	–	22.7%	50.1%	27.2%	–	–	–
Main >60%/25%	>60% insertive	25%	22.7%	50.1%	27.2%	–	Main baseline	10.2%
Main >80%/25%	>80% insertive	25%	22.7%	50.1%	27.2%	–	Main baseline	7.8%
Main 100%/25%	100% insertive	25%	22.7%	50.1%	27.2%	–	Main baseline	5.4%
Main >60%/50%	>60% insertive	50%	22.7%	50.1%	27.2%	–	Main baseline	20.3%
Main >80%/50%	>80% insertive	50%	22.7%	50.1%	27.2%	–	Main baseline	15.5%
Main 100%/50%	100% insertive	50%	22.7%	50.1%	27.2%	–	Main baseline	10.8%
HRS baseline	–	–	54.0%	34.9%	11.1%	–	–	–
HRS	>80% insertive	25%	54.0%	34.9%	11.1%	–	HRS baseline	14.5%
RCS-5 baseline	–	–	22.7%	50.1%	27.2%	5 years	–	–
RCS-5	>80% insertive	25%	22.7%	50.1%	27.2%	5 years	RCS-5 baseline	5.1%
RCS-3 baseline	–	–	22.7%	50.1%	27.2%	3 years	–	–
RCS-3	>80% insertive	25%	22.7%	50.1%	27.2%	3 years	RCS-3 baseline	6.0%

Given the central importance of sexual role in circumcision impacts, we explored multiple models for role based on existing data. For our main model, we use data from the 2008 Peru sentinel surveillance, which asks men about AI role over the previous five years; 22.7% reported exclusive insertivity, 50.1% versatility, and 27.2% exclusive receptivity. It is possible that this sample was biased towards receptive men, who are more likely than other MSM to identify as gay and to be reached through some of the study recruiting venues. We thus also use sexual role proportions from the baseline data of the Peru arm of HPTN-039 (54.0% exclusively insertive, 34.9% versatile, 11.1% exclusively receptive) as one sensitivity analysis (“high role segregation” model, or HRS). This sample, subject to its own sources of bias, had an extremely high proportion of men reporting exclusive insertivity; we expect the two studies to bracket the true population proportions. We run this model for the >80%/25% scenario and compare it to the analogous main model.

Our models thus far assume that men remain in one sexual role category for life. We next explore different assumptions about the rates of role-category-switching (RCS), chosen over a feasible but arbitrary range given the paucity of long-term sexual role data. We consider cases in which men switch sexual role categories on average every 3 or 5 years. We assume a memoryless, stochastic process, implying a geometric distribution of time within a given category. We assume men do not switch directly from exclusively insertive to exclusively receptive or vice versa; any switch is to or from versatile, although men may move among all categories over the lifecourse. We consider this analysis to be exploratory, given the lack of lifecourse data on sexual role in Peru specifically, and return to this point in the Discussion.

Each combination of parameters considered is simulated 10 times in order to assess both the mean and variation in outcomes of interest. We use the latter to calculate theoretical confidence intervals around the mean with the t-distribution, which are plotted in most Figures. These provide a sense of the relative stochasticity of the various outcome measures, and must be interpreted in the light of our modeled population size. Since that population size is arbitrary, and in most cases the questions we are investigating do not have a natural null hypothesis, questions of statistical significance are ill-defined in many cases. Instead, the confidence intervals provide a sense of the natural variation that might be expected in outcomes, and how easy or hard it would be to detect differences between an intervention and control or between two different interventions in MSM populations of this size. We explore this point further in the Discussion.

For all intervention scenarios, our outcome measures include proportion of incident cases averted, proportional reduction in prevalence, and number of cases averted per circumcision, all relative to a baseline of identical behavioral parameters but no MSM-MC intervention, and all measured over 25 years. Cases averted are measured by aggregate counts, not at the individual level, since both the baseline and intervention scenarios are stochastic and different individuals will be infected in each. Proportion of cumulative cases averted at time *x* is thus [(incident infections in baseline model from time 0 to *x* – incident infections in intervention model from time 0 to *x*) / incident infections in baseline model from time 0 to *x*]; other metrics share the same logic. Note that the stochasticity of births and non-AIDS deaths will make the population exposed to possible infection vary across scenarios; however, these phenomena, while stochastic, have the same probabilities in every scenario, and examinations of the runs indicate that the potentially exposed population varies by less than 0.2% on average among scenarios.

We then divide the cases averted (at the group level) between those averted among the men circumcised as part of the intervention (i.e. that would be directly detectable in a follow-up study of the intervention) and those in the rest of the population (which would only be detectable from population surveillance or similar methods, and more difficult to attribute to the intervention). For such a comparison, we must define for our baseline (non-intervention) scenarios a population of men who would have been circumcised if the intervention had been available; this is done separately for each intervention scenario, with the same inclusion criteria and uptake as that scenario, and is also repeated 10 times.

For cases averted per circumcision, we consider both a measure with no discounting, and one with 3% annual discounting on both inputs and outputs. Because we are not explicitly modeling cost, our input is a count of circumcisions; in this case discounting may be thought of as occurring in terms of an implicit cost metric, the average cost of an adult circumcision. This discounting allows for additional comparisons with other published MSM-MC models.

## Results

**Figure 1:**
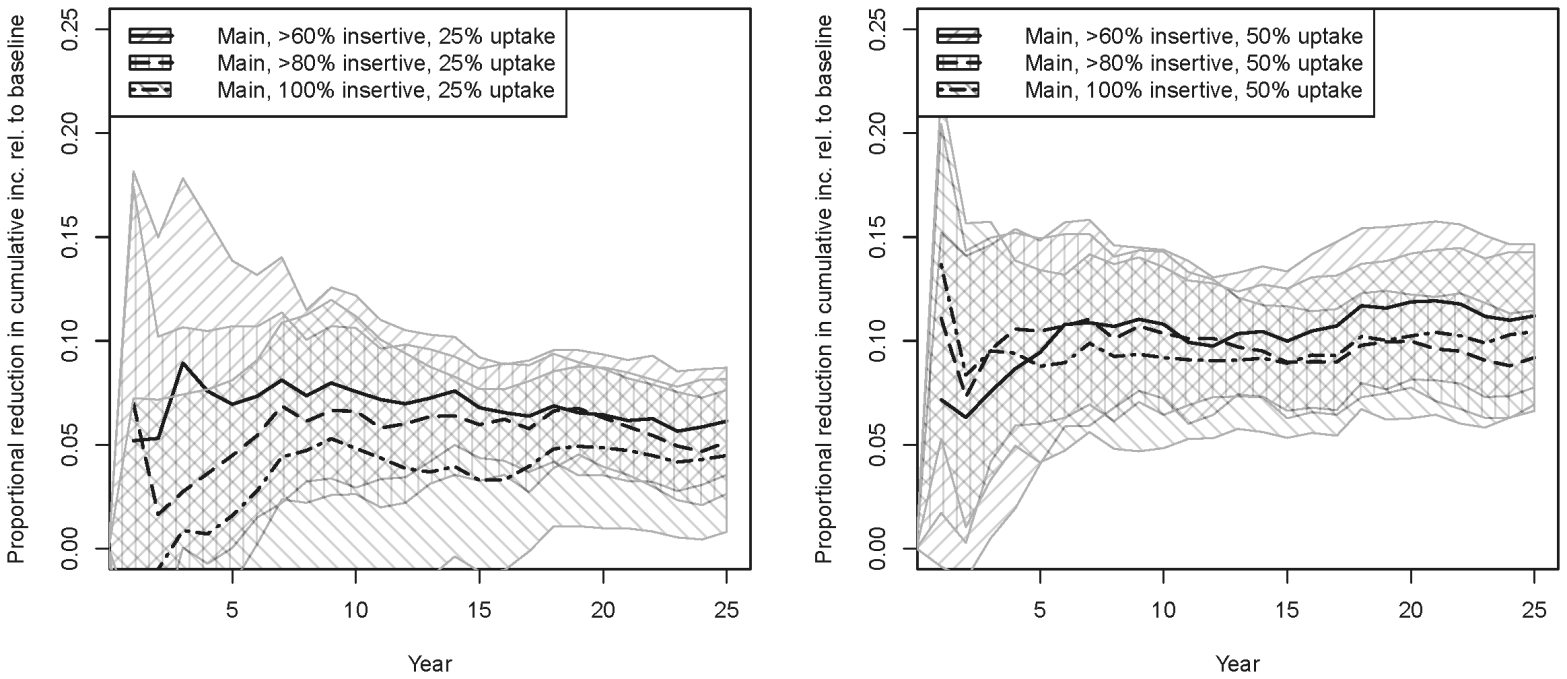
Main intervention scenarios, proportional reduction in incident HIV cases across all MSM relative to main baseline. The proportion of HIV-negative men being circumcised in each scenario is listed in Table 2. Theoretical confidence intervals are generated from the t-distribution based on the variation across 10 simulations, and shown as striped polygons. Figure 1a: 25% uptake; Figure 1b: 50% uptake.

**Figure 2:**
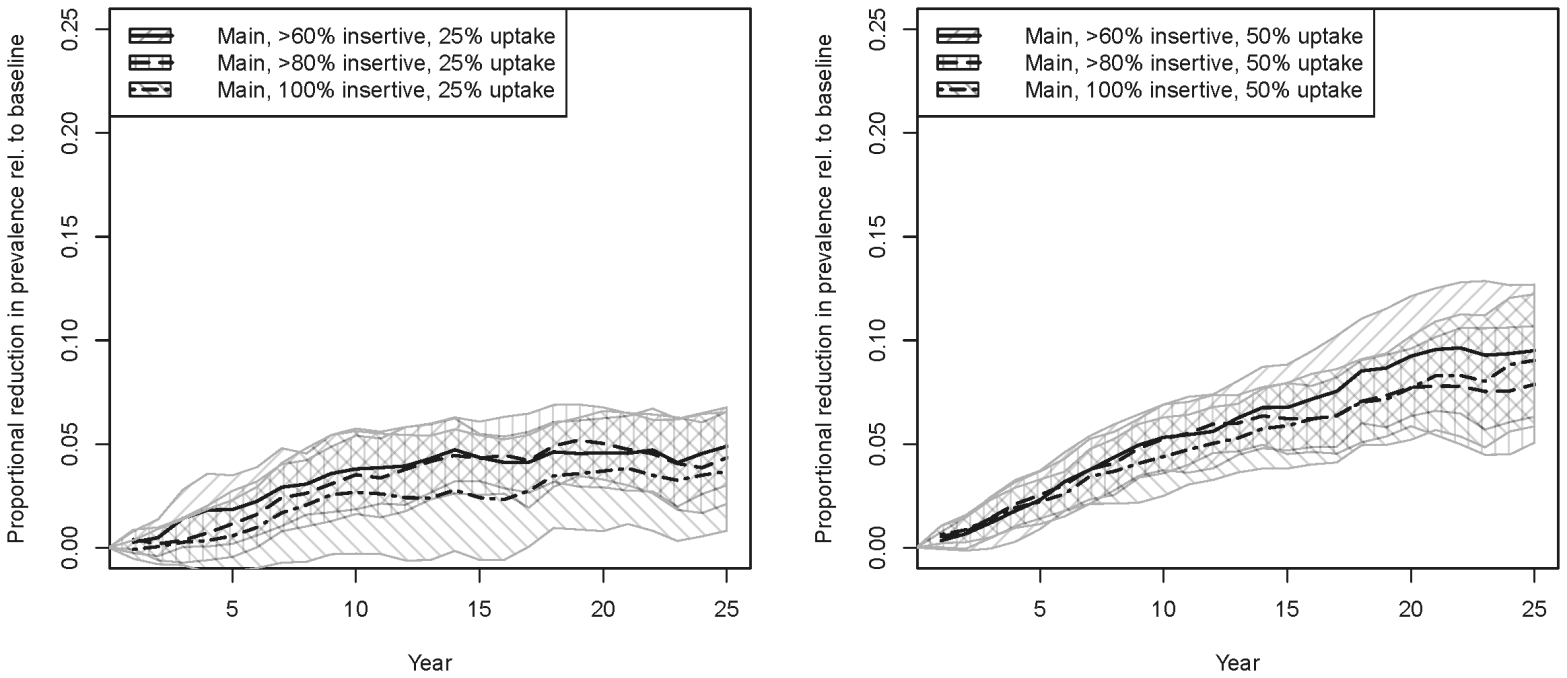
Main intervention scenarios, proportional reduction in HIV prevalence across all MSM. Figure 2a: 25% uptake; Figure 2b: 50% uptake.

Figure 1 shows the proportion of incident cases averted relative to baseline for our six main scenarios (sexual role proportions from sentinel surveillance, no RCS; Figure 1a = 25% uptake, Figure 1b = 50% uptake). Figure 2 shows the analogous graphs for proportionate reductions in prevalence. Uptake has a roughly linear effect on both of these reductions, i.e. point estimates around 5% for 25% uptake and 10% for 50% uptake. Incidence reductions reach these numbers within a few years; prevalence reductions logically take longer to accumulate. The impacts of increasing the eligible population are smaller; limiting to exclusively insertive men is nearly as effective as including all men who are >80% or >60% insertive; indeed, the confidence intervals overlap considerably within each figure, suggesting that the incremental impact of including men less than 100% insertive may indeed be difficult to detect in a population of this size. We extend the simulations to 50 years for two scenarios (inclusion criteria of 100% and >80% insertive, both with 25% uptake; results not shown) to check longer-term dynamics; the former equilibrates at ∼4.5% proportional reduction in incidence and prevalence, and the latter at about ∼5% reduction.

**Figure 3:**
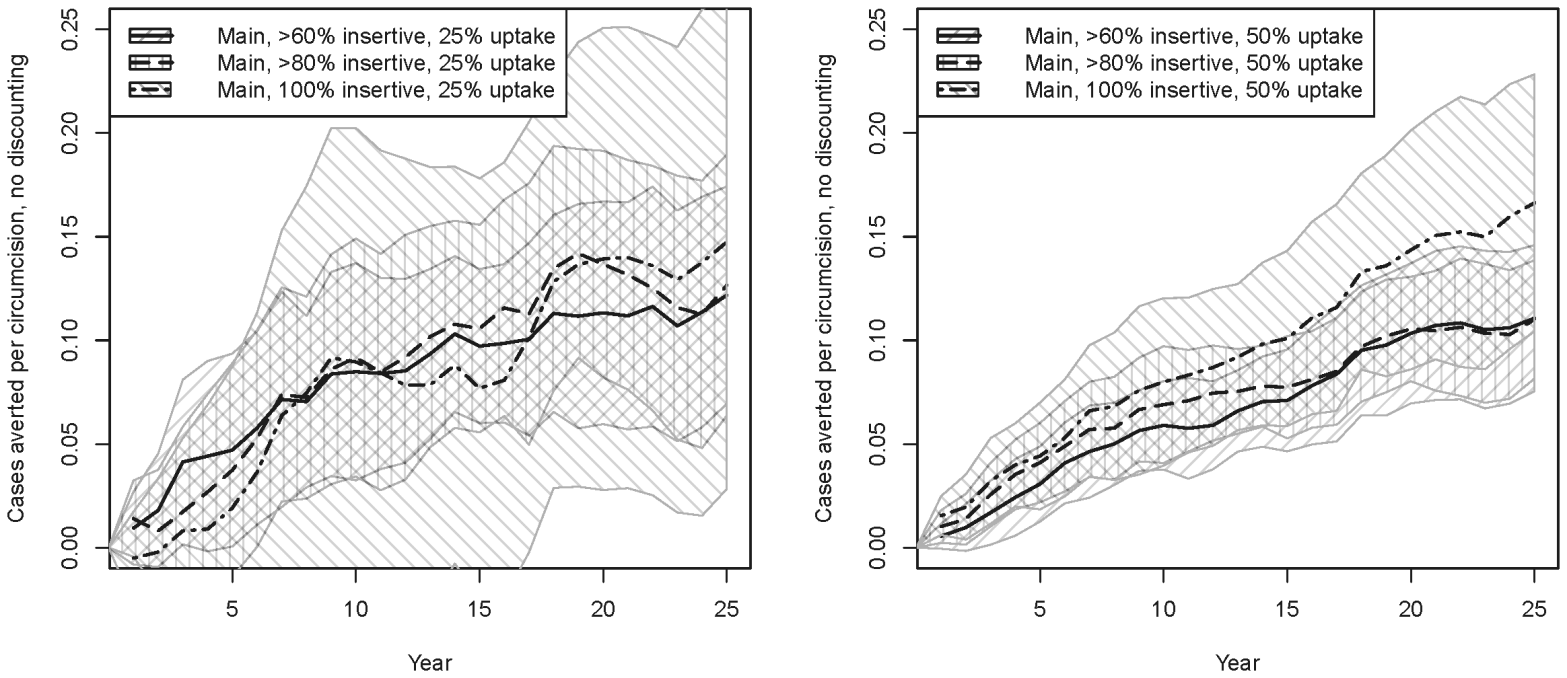
Main intervention scenarios, cumulative cases averted per circumcision, without discounting. Figure 3a: 25% uptake; Figure 3b: 50% uptake.

Figure 3 shows the cases averted per circumcision for these scenarios, without discounting. These begin low due to the large number of circumcisions occurring initially with few immediate cases averted, but rise as both the lifelong protection afforded by the intervention and averted secondary transmissions accumulate. The more restrictive inclusion criteria are the most efficient, reaching a point estimate of ∼0.15 cases averted per circumcision after 25 years, although with considerable variation across runs. This corresponds to a point estimate of ∼6 circumcisions per case averted. The stochasticity for the 100% insertive runs is particularly large, since this metric is a ratio of two stochastic quantities, and the denominator (number of circumcisions) is smallest for this scenario. However, the stochasticity for the 50% uptake runs is smaller than 25% runs, and there we can see the 100% insertive criterion emerging as consistently more efficient than the other two as time passes. When including discounting, the point estimate of ∼0.15 cases averted per circumcision after 25 years reduces to ∼0.12, or about 8 circumcisions per case averted (results not shown).

**Figure 4:**
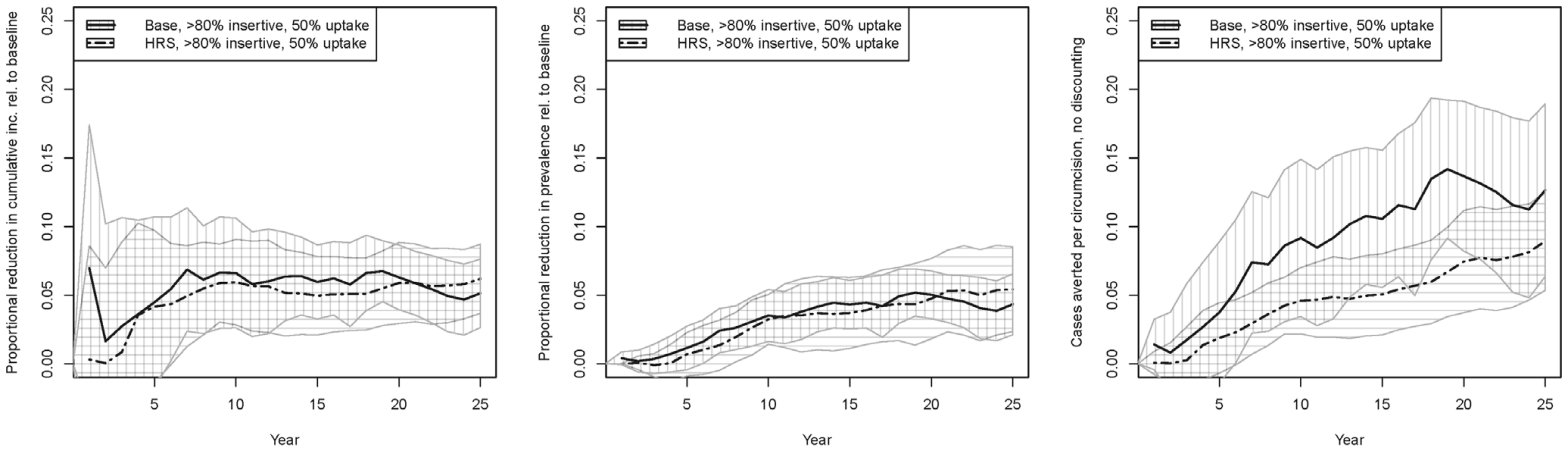
Intervention outcomes for HRS (high role segregation) scenario with 80% insertive threshold /25% uptake, along with analogous main intervention scenario for comparison. Figure 4a: proportional reductions in cumulative incidence: Figure 4b; proportional reductions in prevalence; Figure 4c: cumulative cases averted per circumcision.

We next run our high role segregation (HRS) scenario, using sexual role category proportions from HPTN-039 (54.0% men exclusively insertive compared to 22.7% for previous runs), while continuing to assume no RCS over the life course. We model only the 80% inclusion criterion/25% uptake scenario, and show results in comparison to the analogous main scenario in Figure 4. The impacts on incidence and prevalence appear to be roughly similar, with tightly overlapping confidence intervals. However, because a larger fraction of the population is predominantly insertive in the HRS scenario, more men are eligible for circumcision and more circumcisions are performed; point estimates for cases averted per circumcision are thus ∼30% lower for the HRS scenario, although CIs do again overlap.

**Figure 5:**
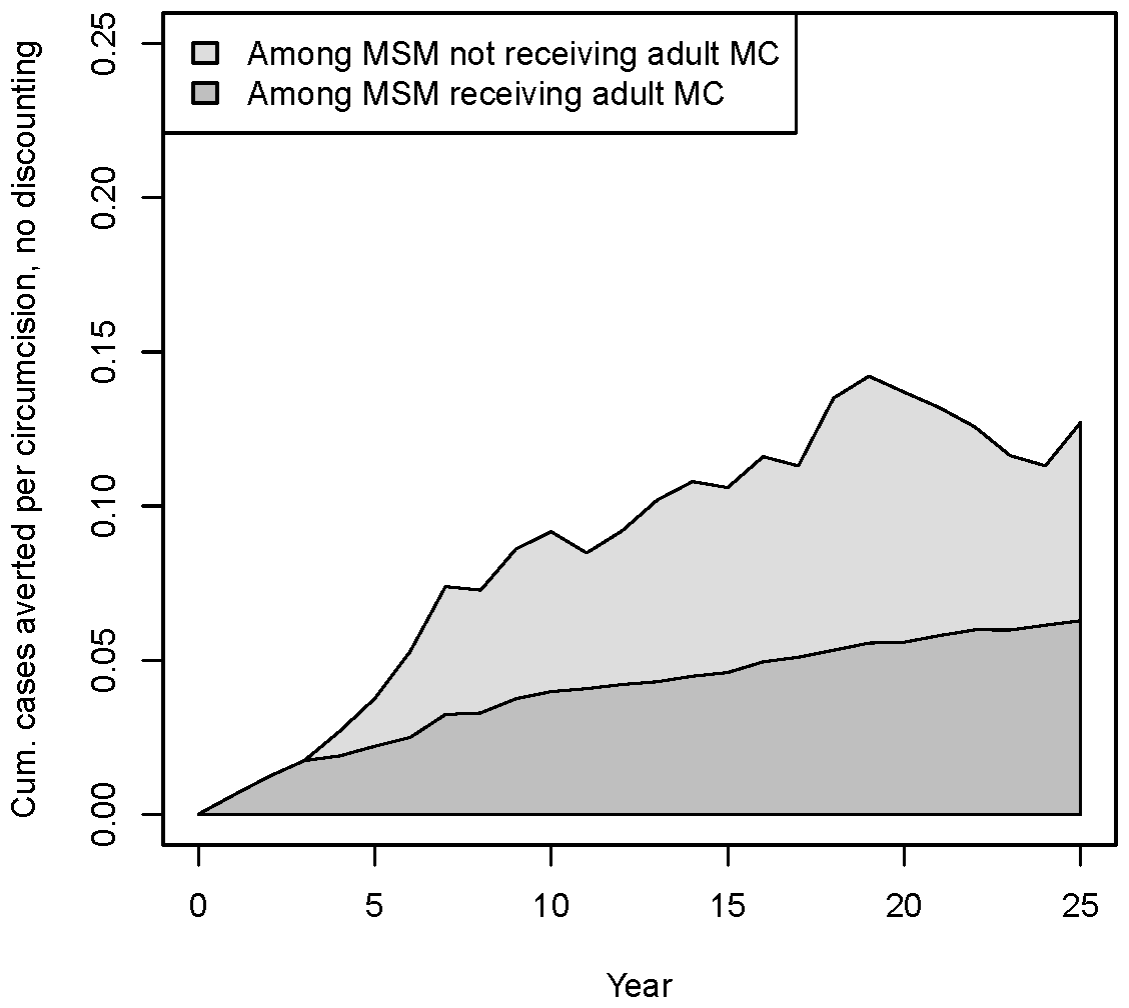
Cumulative cases averted per circumcision for main 80% insertive threshold / 25% uptake scenario, divided between those among men circumcised in the intervention and those in the rest of the MSM population.

We next divide the averted cases into those averted among the men circumcised in the intervention and those not. The latter would be easier to detect by a follow-up study of intervention men to compare their HIV incidence to some pre-intervention baseline of similar men. Figure 5 shows that, in the main 80%/25% scenario, around half of the averted cases would fall into this category, while around half would be in the remainder of the population. Note that the intervention-circumcised men only comprise on average 7.8% of the population, so the fact that they see around half of the averted cases does mean that the intervention effects are disproportionately felt by them; nonetheless, the full impacts of the intervention would be missed if only their outcomes were considered. Confidence intervals are not shown here due to the composite nature of the metric, but the stochasticity is generally much higher in the non-intervention cases averted than the intervention one (as can be indirectly inferred from the greater stability of the latter measure across time than the former in Figure 5). The plot also suggests that the slow increase in overall cases averted per circumcision across the decades that was seen in Figure 3 is due to both the continual protection across the lifecourse afforded to circumcised men, and to additional missed onward transmissions. (Note that for the intervention men, however, we cannot explicitly distinguish these two at the individual level, since they experience both direct and indirect protection).

**Figure 6:**
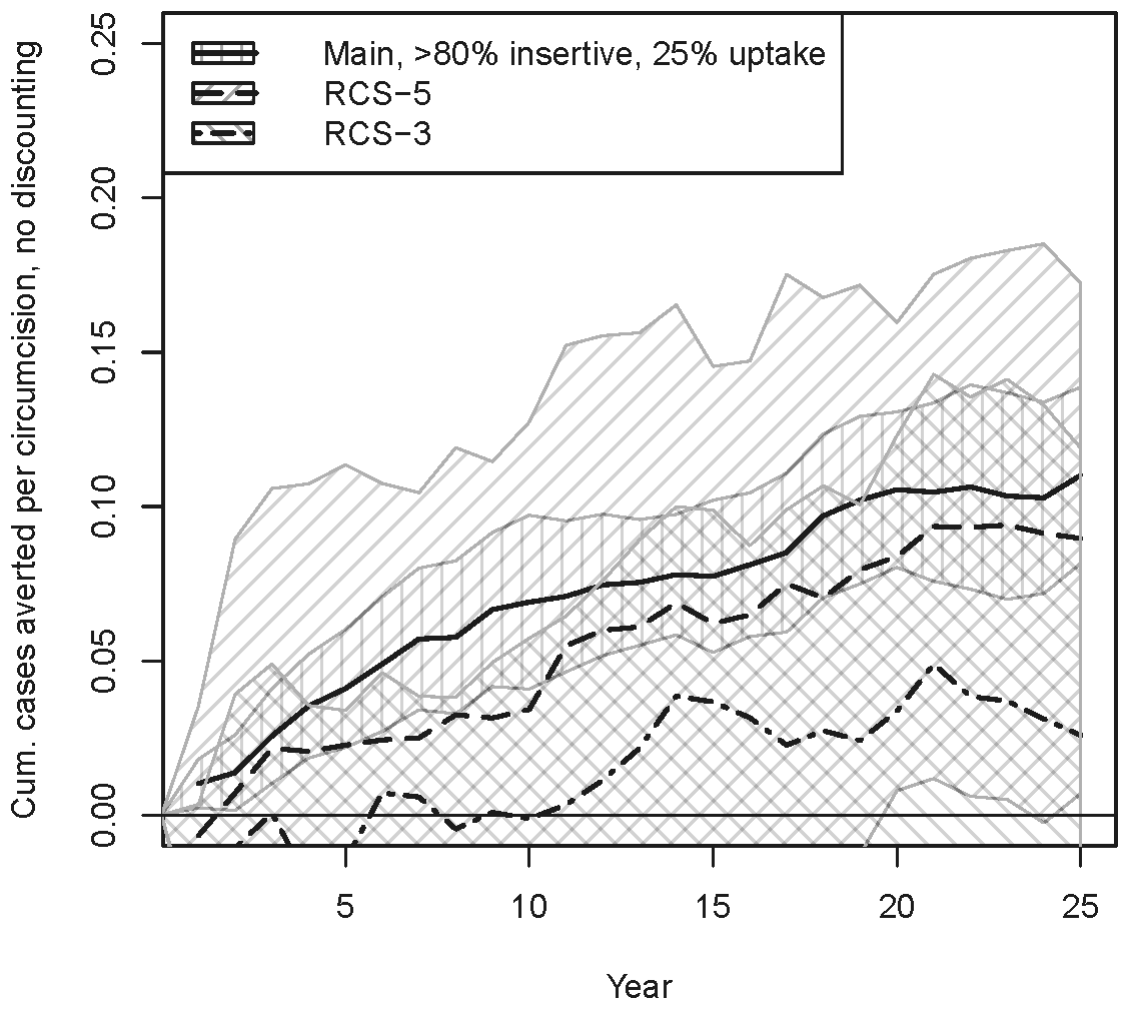
Cumulative cases averted per circumcision for various rates of role category switching (RCS), 80% insertive threshold / 25% uptake.

Next, we return to our baseline sexual role category prevalences, but introduce role category switching (RCS). We consider two rates (men average 5 or 3 years within a given sexual role category), and again focus on the 80%/25% scenario. Figure 6 shows the cumulative cases averted per circumcision (without discounting) for each scenario, with the comparable main scenario with no sexual role switching included for comparison. The more frequent the RCS, the lower the impact of a circumcision intervention, as would be expected. Our point estimate for a 5-year RCS rate is not much lower than for no RCS (∼0.09 after 20 years); for a 3-year RCS rate, the point estimate hovers around ∼0.02–0.05 in later years. However, in both cases the confidence interval extends to 0, i.e. no detectable effect. The low value and high stochasticity of these numbers also makes the estimates of their reciprocal (circumcisions per case averted) unstable; values for the mean run at 25 years is 39 circumcisions per case averted for a 3-year RCS rate, and 11 for 5-year.

**Figure 7:**
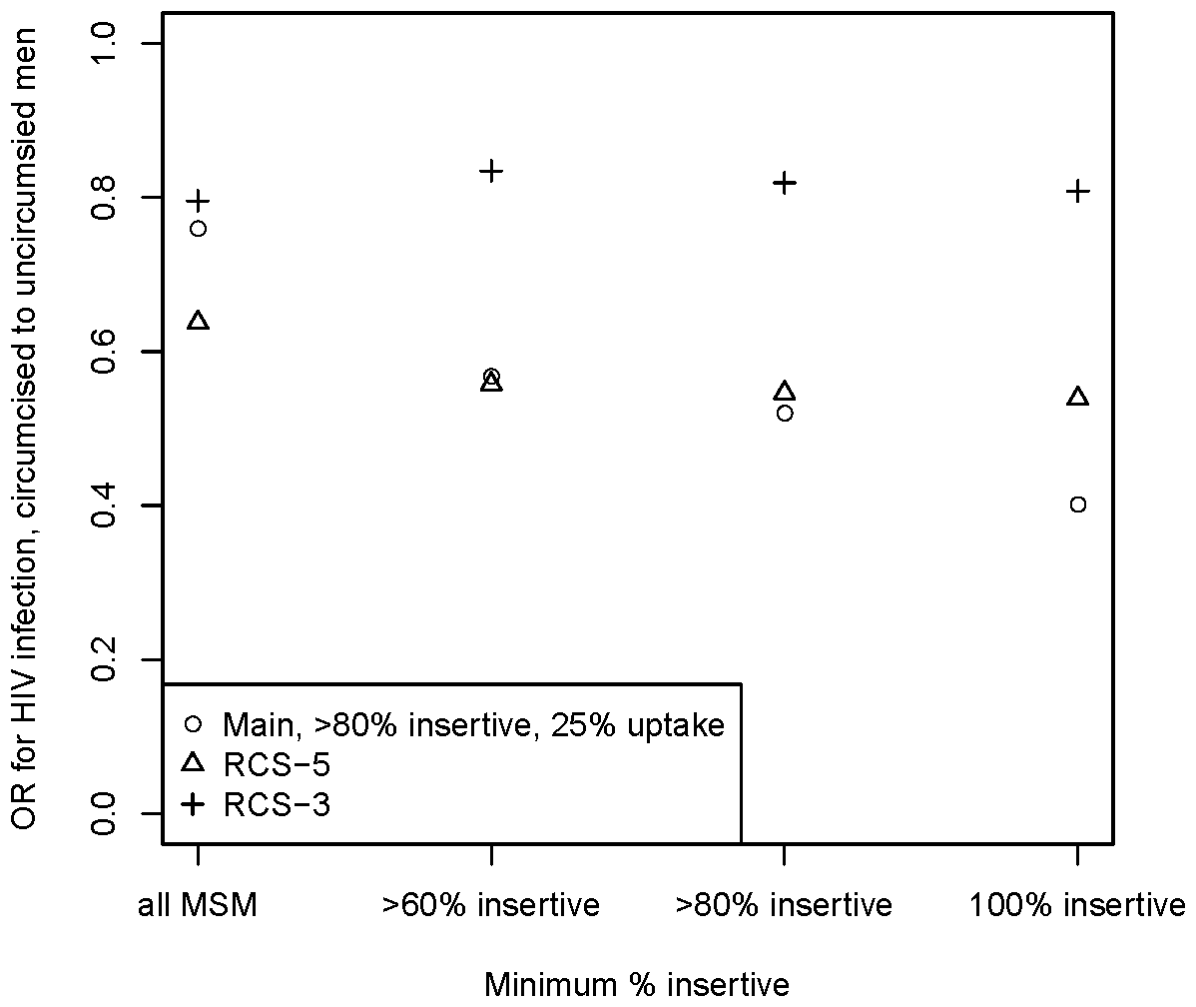
Odds ratios for HIV status by circumcision status observed in the baseline (non-intervention) model.

Finally, to contextualize all previous results, we consider the question: is our assumption that MC has the same proportionate reduction in HIV acquisition for insertive anal and insertive vaginal sex (∼60%) roughly consistent with existing observational studies? We calculate the ORs for HIV status by circumcision status at different insertivity thresholds for our modeled population (without MSM-MC intervention), which is subject to the same spillover effects of circumcision that a natural population would be. Figure 7 shows these values for all MSM and for each definition of predominantly insertive MSM, for our three RCS rates. With little or frequent sexual role switching, ORs are in the range of 0.8 when considering all MSM, near the point estimates and well within the confidence intervals for both meta-analyses and the Peru study. When limiting to exclusively insertive men, point estimates vary more by RCS rate, since the rate of sexual role switching over the life course determines whether men who are currently predominantly insertive were earlier in life as well, and thus their lifetime HIV risk. For the predominantly insertive definition used in the Peru study (≥60%), the point estimates observed in our model are ∼0.56 for the no-RCS and 5-year RCS scenarios; and 0.82 for the frequent (3-year) RCS scenario. Although both numbers are within the confidence interval for the Peru study, the first is much closer to their point estimate (0.31), while still not as strong as it is. We repeated the analysis using RR, with qualitatively similar results.

## Discussion

Our models suggest a rather modest public health impact of a male circumcision intervention for MSM in a setting like Peru, despite the high levels of sexual role segregation, low level of neonatal circumcision, high HIV incidence among MSM, and high levels of potential willingness to be circumcised. Scenarios considered, including coverage up to 50%, suggest that such an intervention is unlikely to avert more than 5–10% of cases over the coming decades. These numbers are similar to those observed in two earlier modeling studies conducted for high-resource settings, although one key difference is that we were able to achieve those reductions with only 25%–50% coverage instead of the 100% coverage included in previous work [21], [22]. Nevertheless, we consider our coverage values to be an upper bound of what is likely achievable for MSM in this setting, such that the overall impact will not be large. Circumcision may, however, be a useful consideration for individual men who are exclusively insertive and who anticipate remaining as such for long periods of time. It is also possible that in a different setting, with different risk practices, sexual mixing, and/or inclusion of other prevention strategies, that a larger impact could be achieved.

We did not conduct an explicit cost-effectiveness analysis. Nevertheless, our main scenarios were more efficient (in terms of circumcisions per case averted) than those considered by Anderson et al. [21], whose analysis found circumcision to be generally cost-effective and sometimes cost-saving. Our higher effectiveness is likely due in part to higher risk behaviors and HIV incidence in this population, as well as high sexual role segregation and low background circumcision prevalence. However, our results, and in particular our measures of circumcisions per case averted, were highly sensitive to assumptions about the frequency with which men change among sexual role categories over the lifecourse. Our point estimates for efficiency approach those of Anderson et al. when men switch among sexual role categories on average every 3 years; however, at this level we also cannot rule out a lack of effect within a population of the size we modeled (10,000 MSM). This sensitivity of circumcision's effects to the frequency of sexual role switching is a particular challenge, given how little data exist on men's long-term role trajectories in any population. Our results suggest that any considerations of an MSM-MC intervention in any population must begin by collecting such data.

Longitudinal data of role switching over time in other settings do exist, but are rare. For instance, early data from the Amsterdam MSM cohort [6] suggested that between two consecutive 6-month periods, among those men continuing to have anal sex, the percentage of insertive, receptive, and versatile men retaining their role category was 69%, 79%, and 71%, respectively. Very few men switched directly from insertive to receptive (4%) or vice versa (3%), consistent with our model. Switches out of versatile were about equal in each direction (15% to insertive, 14% to receptive). However, a higher proportion of insertive men (26%) than receptive men (18%) moved to versatile. In our model, these numbers are equal. Although we do not have similar data for Peru, the age distribution by role in cross-sectional data, where insertivity is more common among younger men, is suggestive of the fact that this pattern is likely to be similar in both settings [13]. If this is indeed the case, then our model may be overstating the potential effectiveness of MSM-MC for any given overall rate of role change, since in reality a higher proportion of insertive men than we model would be progressing to other states.

Our model assumes that the proportional reduction in risk from circumcision is the same for UIAI among MSM as it is for UIVI (∼60%), as do the two previous published papers in their baseline models. There is no direct evidence supporting this assumption, since there has been no circumcision trial among MSM. As a minimum check on its reasonableness, we demonstrated that the assumption is broadly consistent in our modeled population with the two meta-analyses of observational studies that consider HIV prevalence by circumcision status among all MSM and among predominantly insertive MSM. Nevertheless, the estimate remains speculative. It might even be an underestimate, since point estimates for the OR or RR of HIV status by circumcision status among predominantly insertive MSM have been <0.4 in multiple studies conducted in resource-limited settings with a historical pattern of sexual role segregation [19], [20]. One potential reason for the different results by setting in these observational studies is previous history of sexual role switching, since inclusion criteria typically involve role in a bounded time period of the recent past. The observational studies thus provide some suggestive evidence that sexual role switching may be less frequent over the lifecourse in settings like Peru and South Africa than in resource-rich settings. Nevertheless, we reiterate that data on sexual role switching over the lifecourse would be crucial to collect before any MSM-MC intervention.

We included theoretical confidence intervals in our analyses, derived from the variation observed across our ten simulations of each scenario. These represent the amount of stochasticity in outcome that might be expected (with 95% probability) in a population of the size that we modeled. To interpret these, then, it is useful to consider what such a population might represent. The Peruvian census indicates that ∼30% of the nation's population comprises males in our modeled age range [38]. No solid estimate for the percentage of adult males who are MSM exists for Peru. If one believes that figure to be <1%, then the stochasticity in our model may be reflective of the city of Lima; if one believes a figure more like 3%, then the population-size-induced stochasticity would more resemble the metropolitan areas of Trujillo, Arequipa, or Chiclayo (Peru's second- to fourth-largest urban areas, respectively).

One phenomenon that we excluded but which might increase the effectiveness of MSM-MC is behavioral bisexuality among MSM. Female sex partners of such men would be an additional population provided indirect protection by MSM-MC, especially given evidence that exclusively insertive men have the most female partners among MSM [13]. However, these men may also be the least likely to agree to MSM-MC, if it were conducted as a stand-alone intervention without parallel MC interventions among heterosexual males; in this context, opting for adult MC would be a potential signal to partners of same-sex sexual activity. This consideration was part of our motivation to model lower uptake rates than previous studies. In settings where many MSM are behaviorally bisexual and not open about their same-sex sexual activity, MSM–MC will clearly have its greatest uptake—and greatest public health impact—when it occurs in parallel with efforts to increase MC among heterosexual males. Additional challenges for any MSM-MC intervention include the fact that its effects are slow to accumulate (over two decades, given the lifelong protection each circumcision affords), and that about half of the public health effect is not directly detectable, through missed onward transmission.

Elective adult circumcision for MSM is unlikely to achieve a major reduction in new HIV infections in settings like Peru, despite high and relatively stable levels of sexual role segregation and high HIV incidence rates. Additional data on other populations of MSM with high incidence and high role segregation (e.g., parts of Asia and Africa) is needed to determine whether these results are generalizable. MSM-MC should currently be thought of as benefitting an individual, rather than having a substantial public health benefit.
